# Evaluating recommender systems for AI-driven biomedical informatics

**DOI:** 10.1093/bioinformatics/btaa698

**Published:** 2020-08-07

**Authors:** William La Cava, Heather Williams, Weixuan Fu, Steve Vitale, Durga Srivatsan, Jason H Moore

**Affiliations:** Institute for Biomedical Informatics, Department of Biostatistics, Epidemiology and Informatics, University of Pennsylvania, Philadelphia, PA 19104, USA

## Abstract

**Motivation:**

Many researchers with domain expertise are unable to easily apply machine learning (ML) to their bioinformatics data due to a lack of ML and/or coding expertise. Methods that have been proposed thus far to automate ML mostly require programming experience as well as expert knowledge to tune and apply the algorithms correctly. Here, we study a method of automating biomedical data science using a web-based AI platform to recommend model choices and conduct experiments. We have two goals in mind: first, to make it easy to construct sophisticated models of biomedical processes; and second, to provide a fully automated AI agent that can choose and conduct promising experiments for the user, based on the user’s experiments as well as prior knowledge. To validate this framework, we conduct an experiment on 165 classification problems, comparing to state-of-the-art, automated approaches. Finally, we use this tool to develop predictive models of septic shock in critical care patients.

**Results:**

We find that matrix factorization-based recommendation systems outperform metalearning methods for automating ML. This result mirrors the results of earlier recommender systems research in other domains. The proposed AI is competitive with state-of-the-art automated ML methods in terms of choosing optimal algorithm configurations for datasets. In our application to prediction of septic shock, the AI-driven analysis produces a competent ML model (AUROC 0.85±0.02) that performs on par with state-of-the-art deep learning results for this task, with much less computational effort.

**Availability and implementation:**

PennAI is available free of charge and open-source. It is distributed under the GNU public license (GPL) version 3.

**Supplementary information:**

Supplementary data are available at *Bioinformatics* online.

## 1 Introduction

Experimental data are being collected faster than it can be understood across scientific disciplines (Bansal, 2014). The hope of many in the data science community is that widely accessible, open-source artificial intelligence (AI) tools will allow scientific insights from these data to keep abreast of their collection (Olson *et al.*, 2017c). AI is expected to make significant improvements to scientific discovery, human health and other fields in the coming years. One of the key promises of AI is the automation of learning from large sets of collected data. However, at the same time that data collection is outpacing knowledge discovery, methodological improvements from the machine learning (ML) and AI communities are outpacing their dissemination to other fields. As a result, AI and ML continue to have steep learning curves for non-experts, especially for researchers pressed to gain expertise in their own disciplines.

Specialized researchers would benefit greatly from increasingly automated, accessible and open-source tools for AI. With this in mind, we proposed a free and open-source platform called PennAI (http://github.com/EpistasisLab/pennai) (University of Pennsylvania Artificial Intelligence) that allows the non-expert to quickly conduct a ML analysis on their data (Olson *et al.*, 2017c). PennAI uses a web browser-based user interface (UI) to display a user’s datasets, experiments and results, shown inFigure 1. To automate the user’s analysis, PennAI automatically configures and runs supervised learning algorithms catered to the user’s datasets and previous results.

**Fig. 1.**
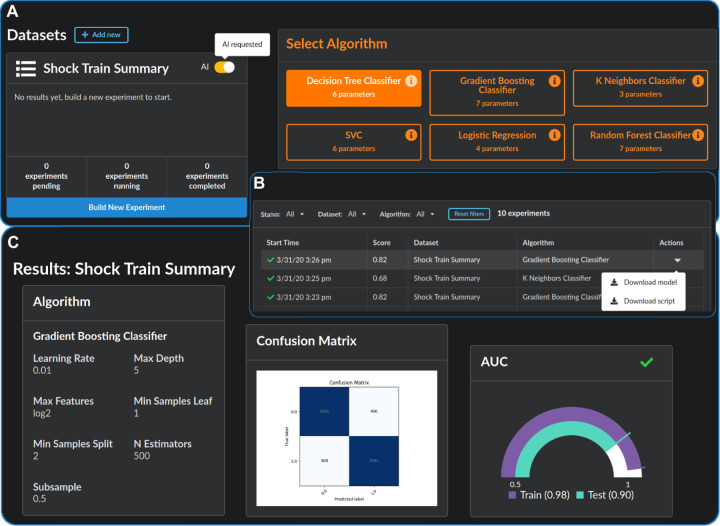
Overview of the UI. (**A**) Users upload datasets and choose a custom experiment (right), or allow the AI to run experiments of its choosing by clicking the AI button (left). (**B**) Experiments are tabulated with configuration and performance information. The user may download scripts to reproduce the experiment in python, or export the fitted model. (**C**) The results page displays experiment information and statistics of the fitted model, including various performance measures [confusion matrix, receiver operating characteristic (ROC) curve, etc.] as well as feature importance scores for the independent variables

In addition to its use as a data science tool, PennAI serves as a test-bed for methods development by AI researchers who wish to develop automated machine learning (AutoML) methods. AutoML is a burgeoning area of research in the ML community that seeks to automatically configure and run learning algorithms with minimal human intervention. A number of different learning paradigms have been applied to this task, and several tools are available to the research community (Feurer *et al.*, 2015, 2018; Hutter *et al.*, 2011; Komer *et al.*, 2014; Kotthoff *et al.*, 2017; Olson *et al.*, 2016) as well as commercially (H2O) (Real, 2018). An AutoML competition has been running since 2015 (http://automl.chalearn.org/) focused various budget-limited tasks for supervised learning (Guyon *et al.*, 2016).

Despite what their name implies, many leading AutoML tools are highly configurable and require coding expertise to operate (Feurer *et al.*, 2015, 2018; Hutter *et al.*, 2011; Komer *et al.*, 2014; Kotthoff *et al.*, 2017; Olson *et al.*, 2016), which leaves a gap for adoption to new users. Furthermore, AutoML tools typically wrap many ML analyses via the automation procedure, obscuring the analysis from the user and preventing user input or guidance. In contrast to these strategies, PennAI interacts with the user through algorithm recommendations, and can learn from both its own analysis and that of the user. This interaction is achieved using a web-based recommender system. We present a detailed background of different AutoML strategies in Supplementary Material. In Table 1, a summary of the similarities and differences between a handful of popular AutoML tools and PennAI is given.

**Table 1.** A comparison of AutoML tool characteristics and PennAI

Tool	Method	Free	Open source	Code-free UI	Learns from user
PennAI	Recommender systems	*✓*	*✓*	*✓*	*✓*
HyperoptSklearn	Bayesian Opt	*✓*	*✓*		
AutoSklearn	Bayesian Opt+metalearning	*✓*	*✓*		
TPOT	Genetic programming	*✓*	*✓*		
H20.AI	Genetic algorithms		*✓*	*✓*	

Recommender systems are well-known inference methods underlying many commercial content platforms, including Netflix (Bennett and Lanning, 2007), Amazon (Smith and Linden, 2017), YouTube (Davidson *et al.*, 2010) and others. There have been limited, yet promising, studies of recommender systems as AutoML approaches (Fusi *et al.*, 2018; Yang *et al.*, 2018). However, to date, there has not been an attempt to compare and contrast different underlying strategies. To fill this gap, we benchmark several state-of-the-art recommender systems in this article, using a large experimental design that tests their use as an AutoML strategy.

Our first aim is to assess the ability of state-of-the-art recommender systems to learn the best ML algorithm and parameter settings for a given dataset over a set number of iterations in the context of previous results. We compare collaborative filtering approaches as well as metalearning approaches on a set of 165 open-source classification problems. We find that the best algorithms (matrix factorization) work well without leveraging dataset metadata, in contrast to other AutoML approaches. We demonstrate the ability of PennAI to outperform Hyperopt and perform on par with AutoSklearn, two popular AutoML tools for Python. Our second goal is to test PennAI in application to predictive modeling in the biomedical context. To this end, we use PennAI to develop predictive models of septic shock using the MIMIC-III (Johnson *et al.*, 2016) critical care database. We find that PennAI is useful for quickly finding models with strong performance, in this instance producing a model with performance on par with effort-intensive, recurrent deep neural networks. We include a sensitivity analysis of the predictive model of septic shock produced by PennAI that lends credence to its predictions.

## 2 Materials and methods

Here, we will briefly describe the UI of PennAI. In Section 2.1, we describe the recommender systems that we benchmark in our experiments for automating the algorithm selection problem. Figure 1 gives an overview of the data science pipeline. Users upload datasets through the interface or optionally by pointing to a path during startup. At that point, users can choose between building a custom experiment (manually configuring an algorithm of their choice) or simply clicking the AI button. Once the AI is requested, the recommendation engine chooses a set of analyses to run. The AI can be configured with different termination criteria, including a fixed number of runs, a time limit, or running until the user turns it off. As soon as the runs have finished, the user may navigate to the results page, where several visualizations of model performance are available (Fig. 1C).

PennAI is available as a docker image that may be run locally or on distributed hardware. Due to its container-based architecture, it is straightforward to run analysis in parallel, both for datasets and algorithms, by configuring the docker environment. For more information on the system architecture, refer to Supplementary Material.

### 2.1 Recommendation system

To use recommender systems as a data science assistant, we treat datasets as users, and algorithms as items. The goal of the AI is therefore as follows: given a dataset, recommend an algorithm configuration to run. Once the algorithm configuration has been run, the result is now available as a rating of that algorithm configuration on that dataset, as if the user had rated the item. This context is beneficial for recommender systems, since normally users only occasionally rate the items they are recommended. We denote this knowledge base of experimental results as $D=\{r_{ad},r_{be},\ldots \}$, where *r_ad_* is the test score, i.e. rating, of algorithm configuration *a* on dataset *d*. In our experiments, the test score is the average 10-fold CV score of the algorithm on the dataset.

With a few notable exceptions discussed below, the recommenders follow this basic procedure:

1. Whenever new experiment results are added to D, update an internal model mapping datasets, *d*, to algorithm configurations, *a*.
2. Given a new recommendation request, generate ${\hat{r}}_{ad}$, the estimated the score of algorithm configuration *a* on dataset *d*. Do this for every *ad* pair that has not already been recommended.
3. Return recommended algorithm configurations in accordance with the termination criterion, in order of best rating to worst.

Note that, the knowledge base D can be populated not only by the AI, but by the user through manual experiments (Fig. 1A) and by the initial knowledge base for PennAI. In production mode, the knowledge base is seeded with approximately 1 million ML results generated on 165 open-source datasets, detailed by Olson *et al.* (2017a). The user may also specify their own domain-specific cache of results.

We benchmark several recommender strategies in the experimental section of this article. Most of these recommenders are adapted from the Surprise recommender library (Hug, 2017). We describe each of them in Supplementary Material. Below, we describe the method we found to be most successful in our experiments: singular value decomposition (SVD).

#### 2.1.1 Singular value decomposition

The SVD recommender is a collaborative filtering method popularized by the top entries to the Netflix challenge (Bennett and Lanning, 2007). Like other top entrants (Bell *et al.*, 2007; Bell and Koren, 2007), SVD is based on a matrix factorization technique that attempts to minimize the error of the rankings via stochastic gradient descent (SGD). Each rating is estimated as

(1) $${\hat{r}}_{ad}=\mu +b_{a}+b_{d}+q_{a}^{T}p_{d}.$$

Here, *μ* is the average score of all datasets across all learners; *b_a_* is the estimated bias for algorithm *a*, initially zero; *b_d_* is the estimated bias for dataset *d*, initially zero; $q_{a}$ is a vector of factors associated with algorithm *a* and $p_{d}$ is the vector of factors associated with dataset *d*, both initialized from normal distributions centered at zero. Ratings are learned to minimize the regularized loss function

(2) $$L=\sum_{r_{ad}\in D}{\left(r_{ad}-{\hat{r}}_{ad}\right)}^{2}+\lambda \left(b_{a}^{2}+b_{d}^{2}+||q_{a}|{|}^{2}+||p_{d}|{|}^{2}\right).$$

One of the attractive aspects of SVD is its ability to learn latent factors of datasets and algorithms ($q_{a}$ and $p_{d}$) that interact to describe the observed experimental results without explicitly defining these factors, as is done in metalearning. A historical challenge of SVD is its application to large, sparse matrices, such as the matrix defined by datasets and algorithms (in our experiments, this matrix is about 1 million elements). SVD recommenders address the computational hurdle using SGD to estimate the parameters of Equation 1 using the existing dataset ratings of algorithms only (Gorrell, 2006). SGD is applied by the following update rules:

(3) $$\begin{array}{c} b_{a}\leftarrow b_{a}+\gamma (e_{ad}-\lambda b_{a})\\ b_{d}\leftarrow b_{d}+\gamma (e_{ad}-\lambda b_{d})\\ q_{a}\leftarrow q_{a}+\gamma (e_{ad}p_{d}-\lambda q_{a})\\ p_{d}\leftarrow p_{d}+\gamma (e_{ad}q_{a}-\lambda p_{d}). \end{array}$$

Here, $e_{ad}=r_{ad}-{\hat{r}}_{ad}$. The learning rate *γ* and regularization parameter *λ* are tunable parameters. To facilitate online learning, the parameters in Equation 3 are maintained between updates to the experimental results, and the number of iterations (epochs) of training is set proportional to the number of new results.

## 3 Experiments

**Fig. 2.**
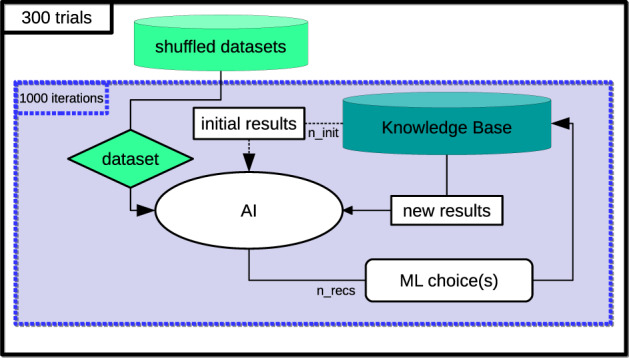
Diagram describing the experimental design. We conduct 300 trials of the experiment, each of which uses a different sampling of datasets. For each trial, 1000 iterations are conducted. The AI (recommender system) is initially trained on *n_init* ML results from the Knowledge Base. Then, each iteration, *n_recs* recommendations are made by the AI for one dataset. These recommended ML configurations are retrieved from the Knowledge Base and used to update the AI for the next iteration

The goal of our experiments is to assess different recommendation strategies in their ability to recommend better algorithm configurations for various datasets as they learn over time from previous experiments. The diagram in Figure 2 describes the experimental design used to evaluate recommendation strategies under PennAI.

*Datasets* We assessed each recommender on 165 open-source classification datasets, varying in size (hundreds to millions of samples) and origin (bioinformatics, economics, engineering, etc.). We used datasets from the Penn Machine Learning Benchmark (PMLB) (Olson *et al.*, 2017b). PMLB is a curated and standardized set of hundreds of open source supervised ML problems from around the web [sharing many in common with OpenML (Vanschoren *et al.*, 2014)]. In previous work, we conducted an extensive benchmarking experiment to characterize the performance of different ML frameworks on these problems (Olson *et al.*, 2017a,b). The benchmark assessed 13 ML algorithms over a range of hyperparameters detailed in Supplementary Table S1 of Supplementary Material on these problems. This resulted in a cache of over 1 million ML results across a broad range of classification problems which we use here to assess the performance of each recommender with a known ranking of algorithms for each dataset. For the experiment in this article, we used a subset of these results consisting of 12 ML algorithms (dropping one of three naïve Bayes algorithms) with 7580 possible hyperparameter configurations.

*Evaluation of recommender systems* The first experiment consisted of 300 repeat trials for each recommender. In each trial, the recommender began with a knowledge base of *n*_init_ experiments that consist of single ML runs on single datasets. For each iteration of the experiment, the recommender was asked to return *n_recs_* recommendations for a randomly chosen dataset. Once the recommendation was made, the 5-fold CV results on training data for the recommended algorithm configurations were fed back to the recommender as updated knowledge. Note that, this experiment mirrors a reinforcement learning experiment in which the actions taken by the recommender (i.e. the recommendations it makes) determine the information it is able to learn about the relationship between datasets and algorithm configurations. To assess the quality of these recommendations without overfitting, we used a separate, hold-out test set performance score for each algorithm configuration on each dataset. We report the scores of each algorithm configuration on these held-out data throughout Section 4. For our experiments, we varied $n_{\text{init}}\in [1,100,10\;000]$ and $n_{\text{recs}}\in [1,10,100]$. These settings control sensitivity to (i) starting with more information and (ii) exploring more algorithm options during learning.

### 3.1 Comparison to state-of-the-art

Based on the results of our first experiment (Section 3), we chose a final configuration for PennAI and benchmarked its performance against two other state-of-the-art AutoML tools: AutoSklearn (Feurer *et al.*, 2015) and HyperoptSklearn (Komer *et al.*, 2014). For AutoSklearn, we restricted the search space to ML configurations to bring it closer to the search spaces of PennAI and HyperoptSklearn that do not use feature preprocessors. Otherwise, we used default settings of both AutoSklearn and HyperoptSklearn. For this comparison, we performed a leave-one-out style analysis, meaning that PennAI is trained on results from all other datasets prior to iteratively making recommendations for a given dataset. This leave-one-out analysis corresponds to the applied case, in which PennAI is deployed with a pretrained recommender and must run experiments for a newly uploaded dataset. For each method, we assessed the generalization performance of the returned model after a given number of evaluations. For AutoSklearn, we assessed its performance as a function of wall-clock run-time, since there was not an apples-to-apples way to control the number of algorithm evaluations.

*Comparison metrics* Since we have the complete results of ML analyses on our experiment datasets, we assessed recommendations in terms of their closeness to the best configuration, i.e. that configuration with the best holdout performance on a dataset among all algorithms in our exhaustive benchmark. Each algorithm configuration is primarily assessed according to its *balanced accuracy* (*BA*), a metric that considers class imbalance by averaging accuracy over classes (Velez *et al.*, 2007). Let the best balanced accuracy on a given dataset be $BA_{d}^{*}$. Then the performance of a recommendation is assessed as the relative distance to the best solution, as:

(4) $$\Delta \text{Balanced}\;{\text{Accuracy}}_{\mathrm{ad}}=\frac{\left({\text{BA}}_{d}^{*}{-\text{BA}}_{\mathrm{ad}}\right)}{{\text{BA}}_{d}^{*}}.$$

In addition to Equation 4, we assessed the AI in terms of the number of datasets for which it is able to identify an ‘optimal’ configuration. Here, we defined ‘optimal’ algorithm configurations to be those that score within some small threshold of the best performance for a dataset. This definition of optimality is of course limited, both by the finite search space defined by the algorithm configuration options and by the choice of threshold (we tried 1% and 5%). Nonetheless, this definition gives a practical indicator of the extent to which AI is able to reach the best known performance.

### 3.2 Illustrative example

In addition to testing different recommendation strategies within PennAI, we applied PennAI to the task of generating a classification model for predicting patient’s risk of septic shock. For this task, we used the MIMIC-III (Johnson *et al.*, 2016) critical care database and preprocessed it according to prior work (Harutyunyan *et al.*, 2019). In addition to the binning process described by Harutyunyan *et al.*, we calculated autocorrelations for each predictor at five different lags to capture time series features. This resulted in a prediction problem with 29 250 training patients, 6284 test patients and 60 dependent variables.

We used the best performing PennAI configuration from our experiments, i.e. the SVD algorithm. We began by allowing the AI to suggest and run 10 experiments. We then manually chose five additional algorithm configurations to run, using default settings in PennAI. These algorithm configurations were chosen to simulate a user who wishes to complement the AI’s choices by choosing different algorithms. The simplest way for a user to do this is to choose default ML configurations, and those are what we report for LogisticRegression, Classification Tree and Random Forest. For the SVC algorithm, we chose two different kernel options. PennAI’s results page was used to validate models on the training set, and to pick a final model for download. We then evaluated this downloaded model on the testing set using PennAI’s model export functionality. Screenshots of this process are shown in Figure 1.

As a point of comparison, we trained a long-term–short-term memory (LSTM) deep learning model using the architecture from Harutyunyan *et al.* (2019). We trained the network for 100 epochs and reported the test results of the final epoch. We also compared to a tuned, penalized logistic regression and to septic shock models from literature (Henry *et al.*, 2015). We discuss these results in Section 4.2.

## 4 Results

**Fig. 3.**
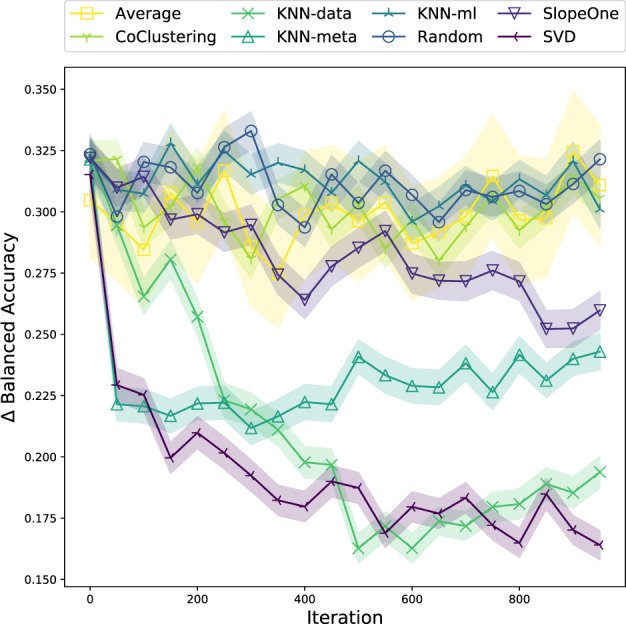
Experiment results for each recommendation strategy with *n*_init_=1, *n*_recs_=10. Each plot shows the median ΔBalanced Accuracy (Equation 4) for 300 trials with error bars denoting 95% confidence intervals. A lower ΔBalanced Accuracy indicates that ML configurations being recommended are closer to the best known configuration

**Fig. 4.**
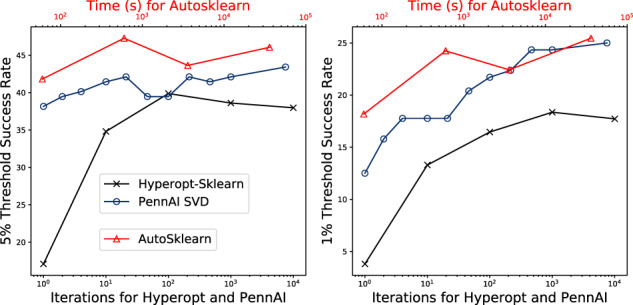
Cumulative success rates across all datasets using PennAI, AutoSklearn and HyperoptSklearn. The success rate is the fraction of datasets for which the recommender has trained an algorithm configuration that achieves a test set balanced accuracy within 1 or 5% of the best performance on that dataset

Results for the PMLB experiment are shown with *n_init_* = 1, *n_recs_* = 10 is shown in Figure 3. The complete results for all settings of *n_init_* and *n_recs_* are given in Supplementary Material. Recommenders are first compared in terms of median ΔBalanced Accuracy (Equation 4) in Figure 3. In Figure 4, we look at the fraction of datasets for which SVD is able to find an optimal configuration under different experiment treatments. In Supplementary Material, we visualize the behavior of a subset of recommenders to gain insight into which algorithms are being selected and how this compares to the underlying distribution of algorithm performance in the knowledgebase.

Let us first focus on the performance results in Figure 3. We find in general that the various recommender systems are able to learn to recommend algorithm configurations that increasingly minimize the gap between the current recommendations and the best performance on each dataset (on hold-out data). SVD performs the best, tending to reach good performance more quickly than the other recommendation strategies. This observation holds across treatments as shown in Supplementary Material (Fig. 2). Across all treatments, KNN-data and KNN-ML tend to be the next best methods. KNN-ML shows a sensitivity to the number of recommendations per iteration, indicating it requires more results to form good clusters. For most experimental treatments, there is a gap between those three methods and the next best recommenders, which vary between SlopeOne, KNN-meta and CoClustering for different settings.

As we expected due to its cold-start strategy, KNN-meta turns out to work well initially, but over time fails to converge on a set of high-quality recommendations. The collaborative filtering recommenders are generally able to learn quickly from few examples compared to the metalearning approach. This difference in performance suggests that the latent similarities between algorithms and between datasets are better learned directly through their performance than through properties of the datasets. Similar results have been reported other domains, for example, in movie recommendations (Pilászy and Tikk, 2009). In that domain, metadata such as genre, director, etc. tends to be less useful for generating recommendations than the user rankings themselves.

### 4.1 Comparison to AutoML

In Figure 4, we compare PennAI with SVD to AutoSklearn and Hyperopt, two widely used AutoML tools. For these comparisons, use the SVD algorithm for PennAI since it is shown to perform the best in terms of ΔBalanced Accuracy in our prior analysis. The side-by-side graphs of Figure 4 show the percent of datasets for which a given method is able to return an algorithm within 5% (left) and 1% (right) of the best performance, as a function of computational effort. The results show AutoSklearn finding best configurations most often for the 5% threshold, and AutoSklearn and PennAI finding best configurations at about the same rate for 1% threshold. In both cases, the performances of AutoSklearn and PennAI within a few percent, whereas HyperoptSklearn tends to under-perform those two methods. Additional comparisons of ΔBalanced Accuracy (Equation 4) for these methods are given in Supplementary Material.

### 4.2 Illustrative example

The screenshots in Figure 1 show the steps in the septic shock model fitting procedure. In Table 2, the fitted models are detailed, including the ML configuration, the 5-fold CV AUROC score, the test set AUROC score and the training time. Based on the 5-fold CV AUROC scores, the gradient boosting model shown in bold was selected and exported from PennAI. This model achieved an AUROC of 0.85 ± 0.02 on the test data, compared to a mean of 0.86 ± 0.02 for the LSTM model trained for 100 epochs. An advantage of the PennAI-generated model is that it took less than 2 min to train, whereas the LSTM model took more than 30 h, using an NVIDIA GeForce GTX 970. Two caveats of this time comparison are that (i) the LSTM is multi-task, i.e. it makes predictions for 25 different phenotypes; and (ii) the results were trained on different hardwares. The PennAI results were generated on a single thread, Intel(R) Core(TM) i7-6950X CPU @ 3.00 GHz. The LSTM results were generated using an NVIDIA GeForce GTX 970 graphics processing unit. Nevertheless, the training time difference of approximately 915x is substantial.

**Table 2.** Area under the receiver operating curve (AUROC) scores for models chosen by the user and AI for predicting shock (Top); control models for comparison (Bottom)

Model	Hyperparameters (Sklearn syntax)	Source	5-fold CV AUROC	Test AUROC	Training time
Logistic regression	Penalty=L2, C=1.0, Dual=False	User	0.84±0.03	0.74±0.03	13 s
	Penalty=L1, C=1e-3, Dual=False	AI	0.81±0.03	0.80±0.02	16 s
KNN classifier	n_neighbors=1, weights=‘distance’	AI	0.68±0.02	0.67±0.03	1 min 1 s
	n_neighbors=7, weights=‘uniform’	AI	0.81±0.03	0.80±0.03	11 min 3 s
	n_neighbors=11, weights=‘uniform’	AI	0.82±0.03	0.81±0.02	11 min 13 s
Support vector	kernel=rbf, C=1.0, degree=3, gamma=0.01	User	0.85±0.02	0.84±0.02	7 min 55 s
	kernel=polynomial, C=1.0, degree=3, gamma=0.01	User	0.82±0.01	0.83±0.02	8 min 43 s
Classification tree	criterion=entropy, max_depth=10	AI	0.75±0.02	0.75±0.03	5 s
	criterion=gini, max_depth=10	user	0.73±0.06	0.59±0.04	6 s
Random forest	criterion=Gini, max_features=‘sqrt’, n_estimators=100	User	0.88±0.06	0.82±0.02	2 min 7 s
	criterion=entropy, max_features=‘log2’, n_estimators=100	AI	0.83±0.06	0.74±0.03	1 min 16 s
Gradient boosting	learning_rate=0.01, max_depth=5, n_estimators=500	AI	**0.90±0.05**	0.85±0.02	1 min 58 s
	learning_rate=0.1, max_depth=5, n_estimators=500	AI	0.89±0.05	0.83±0.02	2 min 25 s
	learning_rate=0.1, max_depth=3, n_estimators=500	AI	0.89±0.05	0.84±0.02	1 min 18 s
	learning_rate=1, max_depth=1, n_estimators=100	AI	0.88±0.06	0.82±0.03	10 s
Tuned logistic regression	Penalty=L2, 10 C values log-spaced in [1e−4, …, 1e4]	Control	0.84±0.02	0.81±0.02	1 min 8 s
*LSTM*	*dim*=*256, depth*=*1, dropout*=*0.3, 100 epochs*	*Harutyunyan et al. (2019)*	–	*0.86*±*0.02*	*30 h 21 min*

*Note*: The model shown in bold was selected based on its 5-fold CV AUROC score, and reports a test AUROC within 1% of the LSTM model (bottom row). Test AUROC is reported using the bootstrapped mean and standard error. Test AUROCs shown in gray are included for evaluation of overfitting, but in practice would not be selected or exported.

**Fig. 5.**
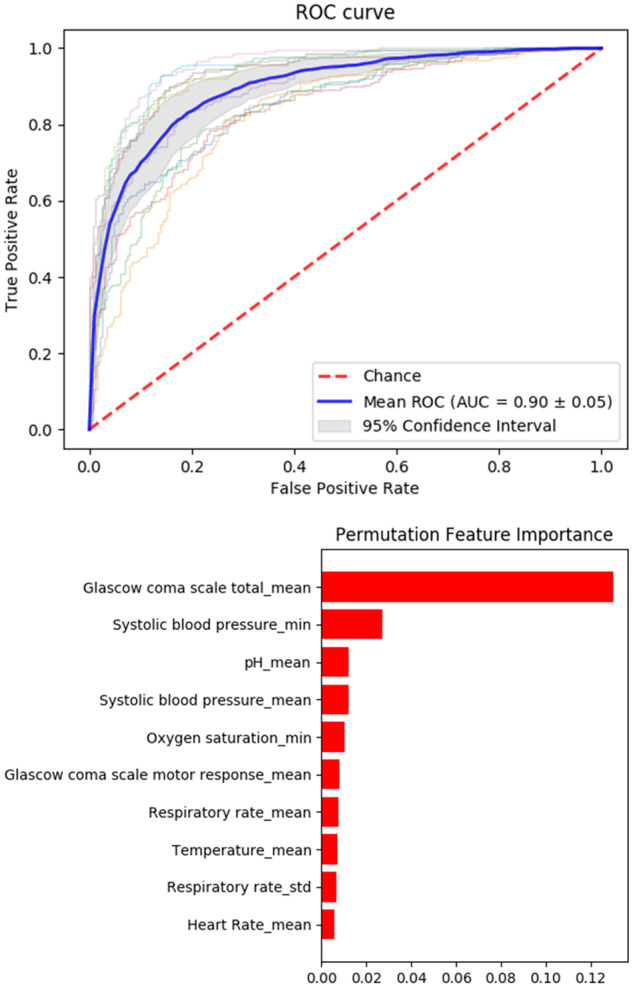
Top: ROC curve for five validation folds of the final septic shock model (Gradient Boosting, bold in Table 2). Bottom: Permutation importance values for dataset features, with larger values indicating features that are more important for prediction

Both model performances are in a similar range to state-of-the-art early detection systems recently deployed to identify septic shock in critical care patients (Henry *et al.*, 2015). Figure 5 shows the cross-validation ROC curves for the gradient boosting model of shock, as well as a sensitivity analysis of the final model using permutation importance (Breiman, 2001). The two most important factors to prediction are the mean Glasgow coma scale rating for the patient and their minimum systolic blood pressure reading. The importance of these two factors lends credence to the model, since they are important for assessing septic shock (Henry *et al.*, 2015). The Glasgow coma scale is an indicator of assessment of the patient’s consciousness and therefore a likely indicator for adverse events. A drop in systolic blood pressure is a tell-tale signature of septic shock and is used as a clinical diagnostic (Singer *et al.*, 2016). Several of the generated models identify these two factors as important for prediction, as shown in Supplementary Material.

## 5 Conclusion

In this article, we propose a data science tool for non-experts that generates increasingly reliable ML recommendations tailored to the needs of the user. The web-based interface provides the user with an intuitive way to launch and monitor analyses, view and understand results, and download reproducible experiments and fitted models for offline use. The learning methodology is based on a recommendation system that can learn from both cached and generated results. We demonstrate through the experiments in this article that collaborative filtering algorithms can successfully learn to produce intelligent analyses for the user starting with sparse data on algorithm performance. We find in particular that a matrix factorization algorithm, SVD, works well in this application area.

PennAI automates the algorithm selection and tuning problem using a recommendation system that is bootstrapped with a knowledgebase of previous results. The default knowledgebase is derived from a large set of experiments conducted on 165 open source datasets. The user can also configure their own knowledgebase of datasets and results catered to their application area. In our application example, we used PennAI with a generic knowledgebase of datasets to successfully train and validate a predictive model of septic shock; we found that PennAI was able to quickly suggest a state-of-the-art model, with little user input. In the future, we hope to further improve PennAI’s ability to handle domain-specific tasks by creating knowledgebases for particular areas such as electronic health records and genetics.

We also hope that PennAI can serve as a testbed for novel AutoML methodologies. In the near term, we plan to extend the methodology in the following ways. First, we plan to implement a focused hyperparameter tuning strategy that can fine-tune the models that are recommended by the AI, similar to AutoSklearn (Feurer *et al.*, 2015) or Hyperopt (Komer *et al.*, 2014). We plan to make this process transparent to the user so that they may easily choose which models to tune and for how long. We also plan to increasingly automate the data preprocessing, which is, at the moment, mostly up to the user. This can include processes from imputation and data standardization to more complex operations like feature selection and engineering.

## Supplementary Material

btaa698_Supplementary_Data
