# Supplementary material for: Evaluating recommender systems for AI-driven biomedical informatics
Source: Bioinformatics. 2020 Aug 7;37(2):250–6. doi: 10.1093/bioinformatics/btaa698 (PMC8055228; doi:10.1093/bioinformatics/btaa698)
Supplement: btaa698_Supplementary_Data [file btaa698_supplementary_data.pdf]

Supplementary Material: Evaluating recommender systems for AI-driven biomedical informatics

William La Cava, Heather Williams, Weixuan Fu, Steve Vitale,
Durga Srivatsan, and Jason H. Moore*

July 24, 2020

1 Background

In this section, we briefly review AutoML methodologies, which are a key component to making data science approachable to new users. We then describe recommender systems, the various methods that have worked in other application areas, and our motivation for applying them to this relatively new area of AutoML.

1.1 Automated Machine Learning

AutoML is a burgeoning area of research in the ML community that seeks to automatically configure and run learning algorithms without human intervention. A number of different learning paradigms have been applied to this task, and tools are available to the research community as well as commercially. A competition around this goal has been running since 2015 [1] focused various budget-limited tasks for supervised learning [1].

A popular approach arising from the early competitions is sequential model-based optimization via Bayesian learning [2], represented by the auto-Weka, AutoSklearn and Hyperopt packages [3, 4, 5]. These tools parameterize the combined problem of algorithm selection and hyperparameter tuning and use Bayesian optimization to select and optimize algorithm configurations.

Auto-sklearn incorporates metalearning into the optimization process [6, 7] to narrow the search space of the optimization process. Metalearning in this context refers to the use of the "metafeatures" of the datasets, such as predictor distributions, variable types, cardinality etc. provide information about algorithm performance that can be leveraged to choose an appropriate algorithm configuration, given these properties for a candidate dataset. Auto-sklearn uses metalearning to narrow the search space of their learning algorithm. In lieu of metalearning, PoSH AutoSklearn [8], an update to AutoSklearn, opted to bootstrap AutoSklearn with an extensive analysis to minimize the configuration space. ML configurations were optimized on a large number of datasets beforehand, and the initial configurations were narrowed to those that performed best over all datasets. This tool effectively replaced metalearning with bootstrapping; our experiments provide additional evidence supporting this strategy.

Another popular method for AutoML is tree-based pipeline optimization tool TPOT [9]. TPOT uses an evolutionary computation approach known as genetic programming to optimize syntax tree representations of ML pipelines. Complexity is controlled via multi-objective search. Benchmark comparisons of TPOT and AutoSklearn show trade-offs in performance for each [10].

There are many commercial tools providing variants of AutoML as well. Many of these platforms do not focus on choosing from several ML algorithms, but instead provide automated ways of tuning specific ones. Google has created AutoML tools as well using neural architecture search [11], a method for configuring the architecture of neural networks. This reflects their historical focus on learning from sequential and structured data like images. H2O uses genetic algorithms to tune the feature engineering pipeline of a user-chosen model. Intel has focused on proprietary gradient boosted ensembles of decision trees [1].

*corresponding author:jhmoore@upenn.edu
[1] http://automl.chalearn.org/

A main paradigm of many AutoML methods is that they wrap several ML analyses and return a single (perhaps ensemble) result, thereby obscuring their analysis from the user. Although this does indeed automate the ML process, it removes the user from the experience. In contrast to these strategies, PennAI uses a recommender system as its basis of algorithm recommendation with the goal of providing a more intuitive and actionable user experience.

There has a line of research utilizing recommender systems for algorithm selection [12, 13, 14, 15]. One recent example is Yang et al. [15], who found that non-negative matrix factorization could be competitive with AutoSklearn for classification. A recent workshop[2] also solicited discussion of algorithm selection and recommender systems, although most research of this nature is interested in tuning the recommendation algorithms themselves [16].

Ultimately, the best algorithm for a dataset is highly subjective: a user must balance their wants and needs, including the accuracy of the model, its interpretability, the training budget and so forth. PennAI's coupling of the recommendation system approach with the UI allows for more user interaction, essentially by maintaining their ability to "look under the hood". The user is able to fully interface with any and all experiments initialized by the AI in order to, for example, interrupt them, generate new recommendations, download reproducible code or extract fitted models and their results. In addition, by making it easy to launch user-chosen experiments, PennAI makes it possible to train the recommender on user-generated results, in addition to its own. By developing PennAI as a free and open-source tool, we also hope to contribute an extensible research platform for the ML and AutoML communities. The code is developed on Github and documents a base recommender class that can be written to the specification of any learning algorithm. We therefore hope that it will serve as a framework for bring real world users into contact with cutting edge methodologies.

1.2 Recommender Systems

Recommender systems are typically used to recommend *items*, e.g. movies, books, or other products, to *users*, i.e. customers, based on a collection of user ratings of items. The most popular approach to recommender systems is collaborative filtering (CF). CF approaches rely on user ratings of items to learn the relationship between similar users and items. In general, CF approaches attempt to group similar users and/or group similar items, and then to recommend similar items to similar users. CF approaches assume, for the most part, that these similarity groupings are implicit in the ratings that users give to items, and therefore can be learned. However, they may be extended to incorporate additional attributes of users or items [17].

Recommenders face challenges when deployed to new users, or in our case, datasets. The new user *cold start problem* [18] refers to the difficulty in predicting ratings for a user with no data by which to group them. With datasets, one approach to this problem is through metalearning. Each dataset has quantifiable traits that can be leveraged to perform similarity comparisons without relying on algorithm performance history. In our experiments we benchmark a recommender that uses metafeatures to derive similarity scores for recommendations, as has been proposed in previous AutoML work [4, 7].

Recommender systems are typically used for different applications than AutoML, and therefore the motivations behind different methods and evaluation strategies are also different. For example, unlike typical product-based recommendation systems, the AI automatically runs the chosen algorithm configurations, and therefore receives more immediate feedback on its performance. Since the feedback is explicitly the performance of the ML choice on the given dataset, the ratings/scores are reproducible, less noisy, and less sparse than user-driven systems. This robustness allows us to measure the performance of each recommendation strategy reliably in varying training contexts. As another example, many researchers have found in product recommendation that the presence or absence of a rating may hold more weight than the rating itself, since users choose to rate or to not rate certain products for non-random reasons [19]. This observation has led to the rise of implicit rating-based systems, such as SVD++ [20], that put more weight on presence/absence of ratings. In the context of AutoML, it is less likely that the presence of results for a given algorithm configuration imply that it will outperform others. Furthermore, the goal of advertising-based, commercial recommendation systems may not be to find the best rating for a user and product, but to promote engagement of the user, vis-a-vis their time spent browsing the website. To this end, recommender systems such as Spotlight [21] are based on the notion of sequence modeling: what is the likelihood of a user interacting

[2] `http://amir-workshop.org/`

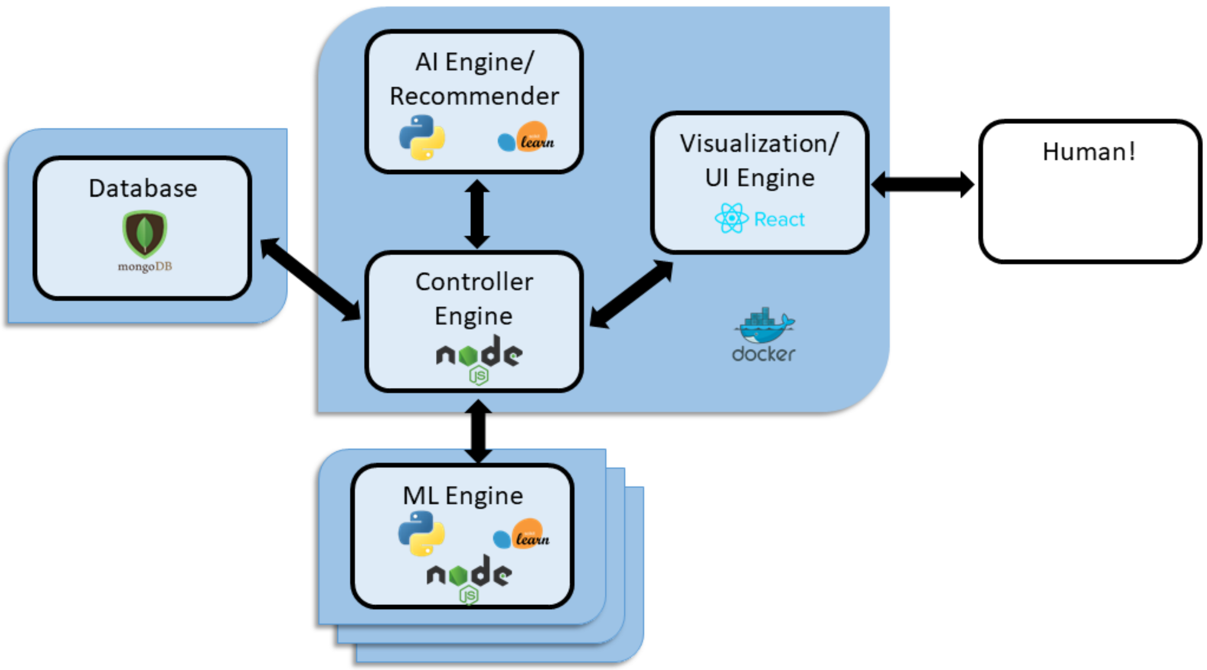

Figure 1: Diagram describing the architecture of PennAI.

with each new content given the sequence of items they have viewed? Sequence-based recommendations may improve the user experience with a data science tool, but we contend that they do not align well with the goals of an approachable data science assistant.

2 System Architecture

PennAI is a multi-component architecture that uses a variety of technologies including Docker[3], Python, Node.js[4], scikit-learn[5], FGLab [6], and MongoDb[7]. The architecture is shown in Figure 1. The project contains multiple docker containers that are orchestrated by a docker-compose file. The central component is the controller engine, a server written in Node.js. This component is responsible for managing communication between the other components using a REST API. A MongoDb database is used for persistent storage. The UI component is a web application written in javascript that uses the React library to create the user interface and the Redux library to manage UI state. The interface supports user interactions including uploading datasets for analysis, requesting AI recommendations for a dataset, manually specifying and running machine learning experiments, and displaying experiment results in an intuitive way. The AI engine is written in Python. As users make requests to perform analysis on datasets, the AI engine will generate new machine learning experiment recommendations and communicate them to the controller engine. The AI engine contains a knowledgebase of previously run experiments, results, and dataset metafeatures that it uses to inform the recommendations it makes. The knowledgebase is bootstrapped with a collection of experiment results generated from the PMLB benchmark datasets. Instructions and code templates are provided to allow easy integration of custom recommendation systems. The machine learning component is responsible for running machine learning experiments on datasets. It has a Node.js server that is used to communicate with the controller engine, and uses Python to execute Scikit-learn experiments on datasets

[3]https://www.docker.com/
[4]https://nodejs.org
[5]http://sklearn.org
[6]https://kaixhin.github.io/FGLab/
[7]https://www.mongodb.com/

and communicate results back to the central server. A PennAI instance can support multiple instances of machine learning engines, enabling multiple experiments to be run in parallel.

3 Benchmarked Recommender Systems

Here, we describe the recommender systems benchmarked in this paper alongside SVD. We refer the interested reader to the Surprise recommender library [22], from which many of these methods were adapted.

3.1 Neighborhood Approaches

We test four different neighborhood approaches to recommending algorithm configurations that vary in their definitions of the neighborhood. Three of these implementations are based the k-nearest neighbors (KNN) algorithm, and the other uses co-clustering. For each of the neighborhood methods, similarity is calculated using the mean squared deviation metric.

In the first and second approach, clusters are derived from the results data directly and used to estimate the ranking of each ML method by computing the centroid of rankings within the neighborhood. Let $N_d^k(a)$ be the k-nearest neighbors of algorithm configuration a that have been run on dataset d. For **KNN-ML**, we then estimate the ranking from this neighborhood as:

$$\hat{r}_{ad} = \frac{\sum_{b \in N_d^k(a)} sim(a,b) \cdot r_{bd}}{\sum_{b \in N_d^k(a)} sim(a,b)} \tag{1}$$

For **KNN-data**, we instead define the neighborhood over datasets, with $N_a^k(d)$ consisting of the k nearest neighbors to dataset d that have results from algorithm a. Then we estimate the rating as:

$$\hat{r}_{ad} = \frac{\sum_{e \in N_a^k(d)} sim(d,e) \cdot r_{ae}}{\sum_{e \in N_a^k(d)} sim(d,e)} \tag{2}$$

For **KNN-data** and **KNN-ML**, the values for k are restricted by the number of existing results for algorithm configurations on datasets. In our experiments, k is restricted to the range $[1, 40]$, and within those values, is set to the number of available results. Note in Equations 1 and 2 that the contribution of each neighbor to the ranking estimate is weighted by its similarity to the algorithm ($sim(a,b)$) or dataset ($sim(d,e)$).

Instead of choosing to define the clusters according to datasets or algorithms, we may define co-clusters to capture algorithms and datasets that cluster together. This is the motivation behind co-clustering [23], the third neighborhood-based approach in this study. Under the **CoClustering** approach, the rating of an algorithm configuration is estimated as:

$$\hat{r}_{ad} = \bar{\mathcal{C}}_{ad} + (\mu_a - \bar{\mathcal{C}}_a) + (\mu_d - \bar{\mathcal{C}}_d) \tag{3}$$

where $\bar{\mathcal{C}}$ is the average rating in cluster $\mathcal{C}$. As Eqn. 3 shows, clusters are defined with respect to a and d together and separately. Co-clustering uses a k-means strategy to define these clusters. We set k equal to the number of algorithms (12) for C_a, and equal to 10 for $\mathcal{C}_d$. It is possible that other values of k would outperform these settings. In case the dataset is unknown, the average algorithm rating, μ_a, is returned instead; likewise if the algorithm configuration is unknown, the average dataset rating μ_d is used. In case neither is known, the global average rating μ is returned.

Finally, we test a metalearning method dubbed **KNN-meta**. In this case, the neighborhood is defined according to metafeature similarity, in the same way as other approaches [6, 7, 4]. We use a set of 45 metafeatures calculated from the dataset, including properties such as average correlation with the dependent variable; statistics describing the mean, max, min, skew and kurtosis of the distributions of each independent variable; counts of types of variables; and so on.

Rather than attempting to estimate ratings of every algorithm, KNN-meta maintains an archive of the best algorithm configuration for each dataset experiment. Given a new dataset, KNN-meta calculates the k nearest neighboring datasets and recommends the highest scoring algorithm configurations from each dataset. KNN-meta sets k to a user-defined parameter n_{recs}, which we vary in our experiments (see the

experiments section of the main paper). KNN-meta has the advantage in cold starts since it does not have to have seen a dataset before to reason about its similarity to other results; it only needs to know how its metafeatures compare to previous experiments. KNN-meta has the limitation, however, that it can only recommend algorithm configurations that have been tried on neighboring datasets. In the case that all of these algorithm configurations have already been recommended, KNN-meta will recommends uniform-randomly from algorithms and their configurations.

3.2 Slope One

Slope one [24] is a simple recommendation strategy that models algorithm performance on a dataset as the average deviation of the performance of algorithms on other datasets with which the current dataset shares at least one analysis in common. To rate an algorithm configuration a on dataset d, we first collect a set $\mathcal{R}_a(d)$ of algorithms that have been trained on d *and* share at least one common dataset experiment with a. We define the shared set of dataset experiments between algorithm a and an algorithm $b \in \mathcal{R}_a(d)$ as $\mathcal{D}_{ab}$. Then the rating is estimated as

$$\hat{r}_{ad} = \mu_d + \frac{1}{|\mathcal{R}_a(d)|} \sum_{b \in \mathcal{R}_a(d)} \left(\frac{1}{|\mathcal{D}_{ab}|} \sum_{e \in \mathcal{D}_{ab}} r_{ae} - r_{be} \right). \tag{4}$$

3.3 Control Recommenders

As a control, we test two baseline algorithms: a random recommender and an average best recommender. The **Random** recommender chooses uniform-randomly among ML methods, and then uniform-randomly among hyperparameters for that method to make recommendations. The **Average** recommender keeps a running average of the best algorithm configuration as measured by the average cross validation (CV) balanced accuracy across experiments. Given a dataset request, the Average recommender recommends algorithm configurations in order of their running averages, from best to worst.

4 Additional Experiment Details

In Table 1, the parameter spaces of each ML algorithm that was used in our experimental analysis is shown. In total, there were 7580 combinations.

4.1 Controlling for Overfitting

We address overfitting in PennAI, and in our experiments, in several ways. All ML analyses are conducted and evaluated using 5- or 10-fold CV. The results page for these analyses (see Fig. 1 of the main paper) include comparisons of training and test scores, with a check mark or x serving as a visual indicator of whether the training score is much better than validation (potential overfitting). Although this alerts the user to overfitting to a certain extent, this does not prevent a model from reporting a high CV score by chance. As the number of analyses (i.e., number of PennAI recommendations) increase, the risk of overfitting to the validation data increases.

To control for overfitting to validation data in deployment and comparison to other models, only a single model should be selected from PennAI for deployment or comparison to other models, and it should be tested on hold-out/test data not used for training or validation/selection. We have designed the tool to support this by providing export scripts for *single models* that include the fitted model as well as template code that the user can use with an external test dataset for final testing or deployment. We have made it implicitly difficult to bulk export all fitted models to discourage new users from violating these practices.

In the illustrative example, we have also followed this procedure - i.e. we chose the single model with the highest 10-fold CV score in PennAI, export it, and then reported its score on holdout test data for performance comparison. It is also for this reason that we focus on one selected model (shown in bold) for test set comparisons in Table 2 of the main paper. However, we have included the other model test results, grayed out, in order to assess overfitting transparently.

Table 1: Analyzed algorithms with their parameters settings. The methods and parameters are named according to Scikit-learn nomenclature[25].

**Algorithm name**	**Parameter**	**Values**
AdaBoostClassifier	learning_rate<br>n_estimators	[0.01, 0.1, 0.5, 1.0, 10.0, 50.0, 100.0]<br>[10, 50, 100, 500, 1000]
BernoulliNB	alpha<br>fit_prior<br>binarize	[0.0, 0.1, 0.25, 0.5, 0.75, 1.0, 5.0, 10.0, 25.0, 50.0]<br>[‘true’, ‘false’]<br>[0.0, 0.1, 0.25, 0.5, 0.75, 0.9, 1.0]
DecisionTreeClassifier	min_weight_fraction_leaf<br>max_features<br>criterion	[0.0, 0.05, 0.1, 0.15, 0.2, 0.25, 0.3, 0.35, 0.4, 0.45, 0.5]<br>[0.1, 0.25, 0.5, 0.75, ‘log2’, None, ‘sqrt’]<br>[‘entropy’, ‘gini’]
ExtraTreesClassifier	n_estimators<br>min_weight_fraction_leaf<br>max_features<br>criterion	[10, 50, 100, 500, 1000]<br>[0.0, 0.05, 0.1, 0.15, 0.2, 0.25, 0.3, 0.35, 0.4, 0.45, 0.5]<br>[0.1, 0.25, 0.5, 0.75, ‘log2’, None, ‘sqrt’]<br>[‘entropy’, ‘gini’]
GradientBoostingClassifier	loss<br>learning_rate<br>n_estimators<br>max_depth<br>max_features	[‘deviance’]<br>[0.01, 0.1, 0.5, 1.0, 10.0]<br>[10, 50, 100, 500, 1000]<br>[1, 2, 3, 4, 5, 10, 20, 50, None]<br>[‘log2’, ‘sqrt’, None]
KNeighborsClassifier	n_neighbors<br>weights	[1, 2, . . ., 25]<br>[‘uniform’, ‘distance’]
LogisticRegression	C<br>penalty<br>fit_intercept<br>dual	[0.5, 1.0, . . ., 20.0]<br>[‘l2’, ‘l1’]<br>[‘true’, ‘false’]<br>[‘true’, ‘false’]
MultinomialNB	alpha<br>fit_prior	[0.0, 0.1, 0.25, 0.5, 0.75, 1.0, 5.0, 10.0, 25.0, 50.0]<br>[‘true’, ‘false’]
PassiveAggressiveClassifier	C<br>loss<br>fit_intercept	[0.0, 0.001, 0.01, 0.1, 0.5, 1.0, 10.0, 50.0, 100.0]<br>[‘hinge’, ‘squared_hinge’]<br>[‘true’, ‘false’]
RandomForestClassifier	n_estimators<br>min_weight_fraction_leaf<br>max_features<br>criterion	[10, 50, 100, 500, 1000]<br>[0.0, 0.05, 0.1, 0.15, 0.2, 0.25, 0.3, 0.35, 0.4, 0.45, 0.5]<br>[0.1, 0.25, 0.5, 0.75, ‘log2’, None, ‘sqrt’]<br>[‘entropy’, ‘gini’]
SGDClassifier	loss<br>penalty<br>alpha<br>learning_rate<br>fit_intercept<br>l1_ratio<br>eta0<br>power_t	[‘hinge’, ‘perceptron’, ‘log’, ‘squared_hinge’, ‘modified_huber’]<br>[‘elasticnet’]<br>[0.0, 0.001, 0.01]<br>[‘constant’, ‘invscaling’]<br>[‘true’, ‘false’]<br>[0.0, 0.25, 0.5, 0.75, 1.0]<br>[0.01, 0.1, 1.0]<br>[0.0, 0.1, 0.5, 1.0, 10.0, 50.0, 100.0]
SVC	C<br>gamma<br>kernel<br>degree<br>coef0	[0.01]<br>[0.01]<br>[‘poly’]<br>[2, 3]<br>[0.0, 0.1, 0.5, 1.0, 10.0, 50.0, 100.0]

In our experiments comparing recommender systems and comparing to state-of-the-art methods, we also control for overfitting by maintaining separate, hold-out test data. Prior to the experiments, each dataset is partitioned into training and testing data. The training data is then further partitioned for 10-fold cross validation. After a given number of iterations, the model with the best 10-fold CV score is tested on holdout data to generate our performance comparisons. As a result, the experiments are designed to capture whether methods overfit with increasing iterations. However, for the most part (see Figure 2 and 3), we don’t see

serious overfitting in the PennAI experiments.

5 Additional Results

Fig. 2 shows the extended experimental comparisons of different recommender systems under different treatments. For a smaller n_{recs}, we observe that the confidence intervals are wider, as we would expect given that fewer algorithm configurations are being evaluated. As the number of initial results in the knowledgebase (n_{init}) increases, the initial recommendations tend to have lower error for all but the random recommender. For most treatments, most of the recommendation strategies exhibit learning; i.e., they produce recommendations that are closer to the best algorithm configuration as they recommend and update over successive iterations. The SVD algorithm, which we choose as the default PennAI recommender in subsequent experiments, is consistently the best performing algorithm across treatments. With large values of n_{recs} and n_{init}, we see the recommendations begin to perform worse in later iterations. This worsening can be due to two factors: 1), overfitting, as described in Section 4.1; and 2), the constraint that recommendations be given for algorithm configurations not yet run on a given dataset. In other words, for a given dataset, if the best algorithm configuration(s) have already have been recommended, subsequent recommendations are expected to be worse. More work is needed to isolate the contribution of these two potential sources of this observation.

The plots in Fig. 4 show the frequency with which SVD, KNN-ML, and SlopeOne recommend different algorithms in comparison to the frequency of top-ranking algorithms by type (the top left plot). Here we see that SVD gradually learns to recommend the top five algorithms in approximately the same ranking as they appear in the knowledgebase. This lends some confidence to the relationship that SVD has learned between algorithm configurations and dataset performance.

5.1 Comparison to AutoML

Fig. 3 considers the same results as Fig. 4 in the main paper but without a threshold, instead showing the average reduction in ΔBalanced Accuracy across datasets for each AutoML method. By this metric, AutoSklearn performs the best, although its performance is not significantly different than that of PennAI, as observed by the overlap of their confidence intervals.

5.2 Illustrative Example

In addition to the main illustrative example results, in Fig. 5 we show permutation feature importance scores for all 15 models from Table 2 of the main paper. Importances are measured by permutation tests [26], and on the right side of Fig 5, we summarize each variable's importance by showing a weighted average of its importances in each model, where the weight is the mean test AUROC value for that model. Overall, the models support the importances of the final gradient boosting model of septic shock, namely that the mean Glasgow coma scale rating for the patient and their minimum systolic blood pressure are the most important factors for prediction.

References

[1] Isabelle Guyon, U Paris-Saclay, Hugo Jair Escalante, Sergio Escalera, U Barcelona, Damir Jajetic, James Robert Lloyd, and Nuria Macia. A brief Review of the ChaLearn AutoML Challenge:. *JMLR Workshop and Conference Proceedings*, 64:21–30, 2016. ZSCC: NoCitationData[s0].

[2] Frank Hutter, Holger H. Hoos, and Kevin Leyton-Brown. Sequential Model-Based Optimization for General Algorithm Configuration. In Carlos A. Coello Coello, editor, *Learning and Intelligent Optimization*, Lecture Notes in Computer Science, pages 507–523. Springer Berlin Heidelberg, 2011.

[3] Lars Kotthoff, Chris Thornton, Holger H. Hoos, Frank Hutter, and Kevin Leyton-Brown. Auto-WEKA 2.0: Automatic model selection and hyperparameter optimization in WEKA. *The Journal of Machine Learning Research*, 18(1):826–830, 2017.

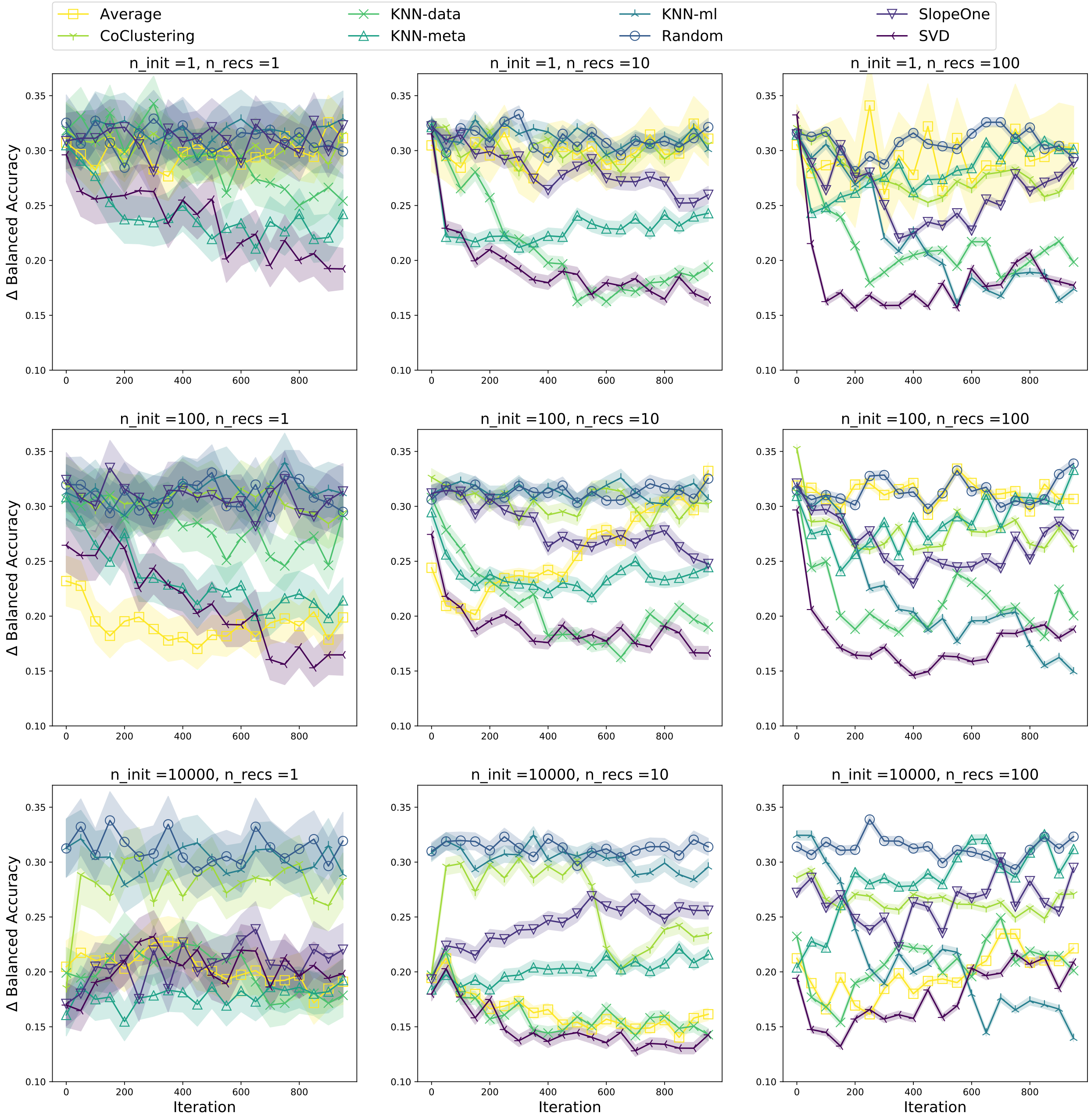

Figure 2: Experiment results for each recommendation strategy. Each plot shows the median Δ Balanced Accuracy for 300 trials with error bars denoting 95% confidence intervals. A lower Δ Balanced Accuracy indicates that ML configurations being recommended are closer to the best known configuration. From left to right, the number of recommendations made per dataset increases; from top to bottom, the number of experiments in the initial knowledgebase increases.

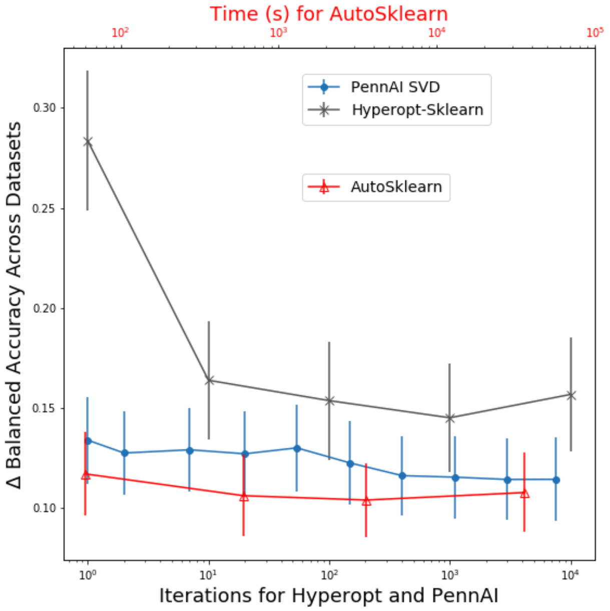

Figure 3: ΔBalanced Accuracy across all datasets, using PennAI, AutoSklearn, and HyperOptSklearn. Error bars indicate the 95% confidence intervals.

[4] Matthias Feurer, Aaron Klein, Katharina Eggensperger, Jost Springenberg, Manuel Blum, and Frank Hutter. Efficient and robust automated machine learning. In *Advances in Neural Information Processing Systems*, pages 2962–2970, 2015.

[5] Brent Komer, James Bergstra, and Chris Eliasmith. Hyperopt-sklearn: automatic hyperparameter configuration for scikit-learn. In *ICML workshop on AutoML*, 2014.

[6] Pavel B. Brazdil, Carlos Soares, and Joaquim Pinto Da Costa. Ranking learning algorithms: Using IBL and meta-learning on accuracy and time results. *Machine Learning*, 50(3):251–277, 2003.

[7] Pavel Brazdil, Christophe Giraud Carrier, Carlos Soares, and Ricardo Vilalta. *Metalearning: Applications to Data Mining*. Springer Science & Business Media, November 2008.

[8] Matthias Feurer, Katharina Eggensperger, Stefan Falkner, Marius Lindauer, and Frank Hutter. Practical automated machine learning for the automl challenge 2018. In *International Workshop on Automatic Machine Learning at ICML*, 2018.

[9] Randal S. Olson, Nathan Bartley, Ryan J. Urbanowicz, and Jason H. Moore. Evaluation of a tree-based pipeline optimization tool for automating data science. In *Proceedings of the Genetic and Evolutionary Computation Conference 2016*, pages 485–492. ACM, 2016.

[10] Adithya Balaji and Alexander Allen. Benchmarking Automatic Machine Learning Frameworks. *arXiv:1808.06492 [cs, stat]*, August 2018. arXiv: 1808.06492.

[11] Esteban Real. Using Evolutionary AutoML to Discover Neural Network Architectures, March 2018.

[12] David Stern, Horst Samulowitz, Ralf Herbrich, Thore Graepel, Luca Pulina, and Armando Tacchella. Collaborative expert portfolio management. In *Twenty-Fourth AAAI Conference on Artificial Intelligence*, 2010.

[13] Mustafa Mısır and Michèle Sebag. Alors: An algorithm recommender system. *Artificial Intelligence*, 244:291–314, 2017.

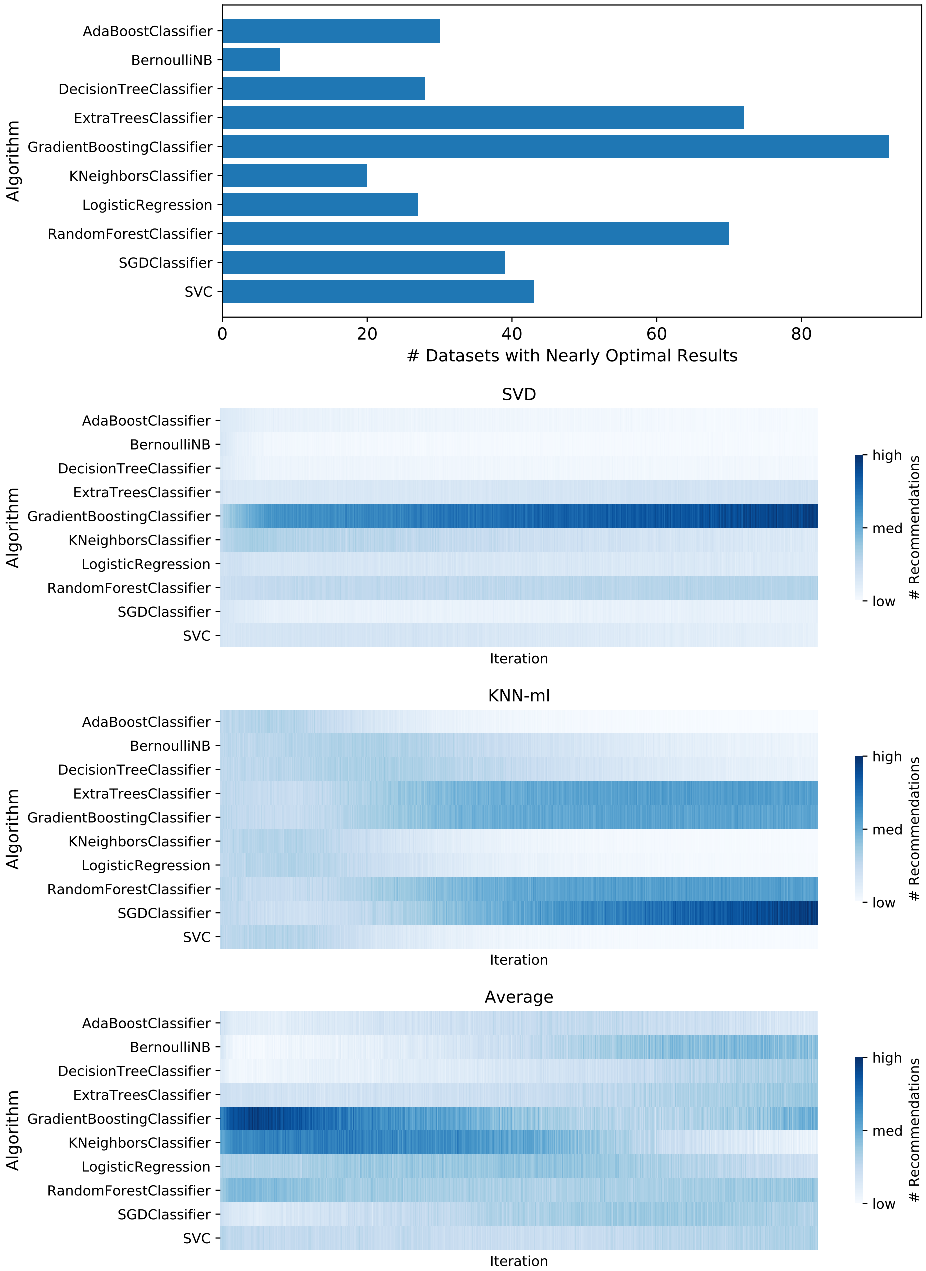

Figure 4: Heatmaps of three different recommendation strategies on the PMLB benchmark showing how often each ML was recommended each iteration. These plots show the experiment treatment with $n_{recs} = 10$ and $n_{init} = 100$. The top figure shows the number of datasets for which each ML algorithm has a configuration that is within 1% of the best performance on that dataset. The second figure shows SVD recommendations; over several iterations, it learns to approximate the frequency distribution of best ML models, i.e. GradientBoostingClassifier, followed by RandomForestClassifier and ExtraTreesClassifier. The third plot shows KNN-ml recommendations; in this case, SGDClassifier ends up being recommended more often than is supported by its benchmark performance. The final plot shows the performance of Average recommender; the distribution of algorithm recommendations is more wide, and tends to drift away from the best algorithm choices after several iterations.

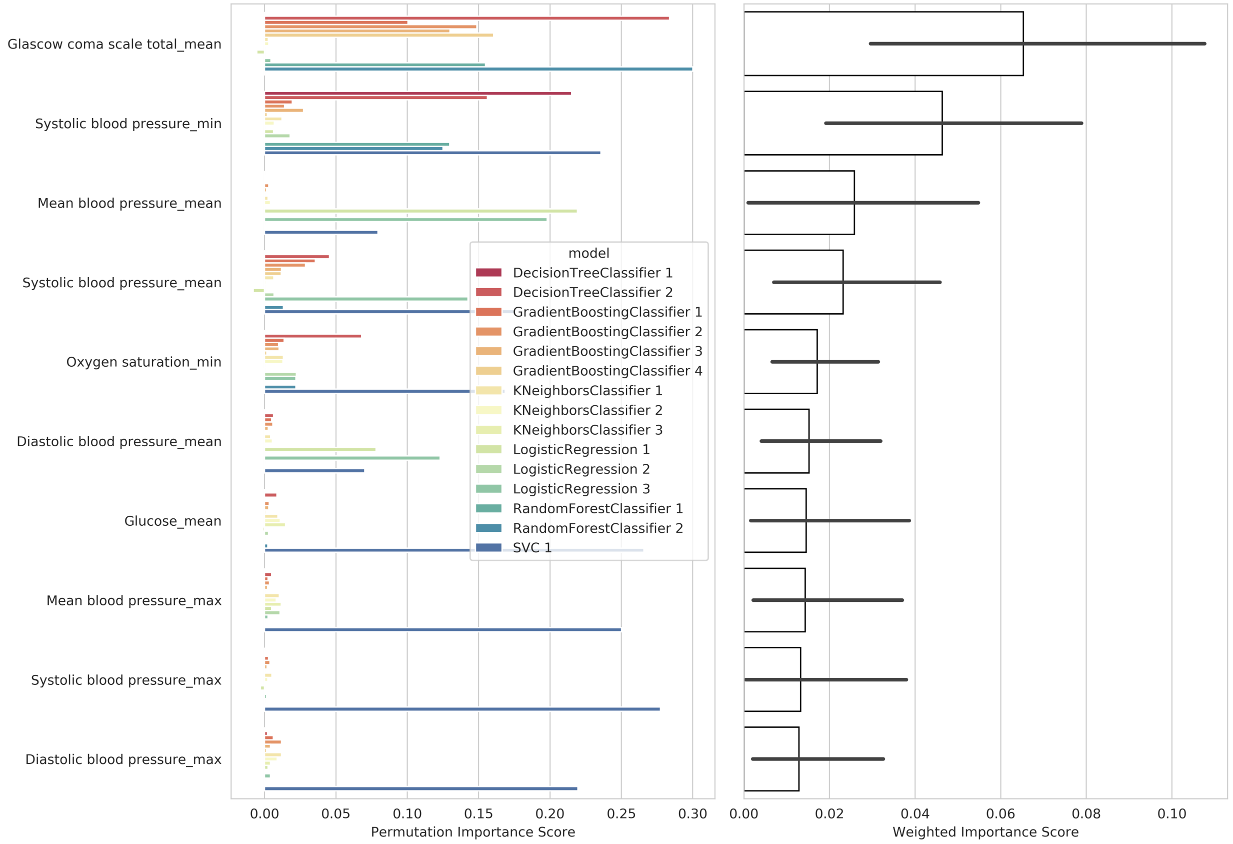

Figure 5: Summary of permutation importance scores for all models trained on the septic shock prediction task. The ten features with the largest average weighted importance scores are shown. Left: importance scores for each separate model; Right: importance score distribution weighted by test set AUROC scores.

[14] Nicolo Fusi, Rishit Sheth, and Melih Elibol. Probabilistic matrix factorization for automated machine learning. In *Advances in Neural Information Processing Systems*, pages 3348–3357, 2018.

[15] Chengrun Yang, Yuji Akimoto, Dae Won Kim, and Madeleine Udell. OBOE: Collaborative Filtering for AutoML Initialization. *arXiv:1808.03233 [cs, stat]*, August 2018. arXiv: 1808.03233.

[16] Tiago Cunha, Carlos Soares, and André C. P. L. F. de Carvalho. Metalearning and Recommender Systems: A literature review and empirical study on the algorithm selection problem for Collaborative Filtering. *Information Sciences*, 423:128–144, January 2018.

[17] Lyle H. Ungar and Dean P. Foster. Clustering methods for collaborative filtering. In *AAAI workshop on recommendation systems*, volume 1, pages 114–129, 1998.

[18] Andrew I. Schein, Alexandrin Popescul, Lyle H. Ungar, and David M. Pennock. Methods and metrics for cold-start recommendations. In *Proceedings of the 25th annual international ACM SIGIR conference on Research and development in information retrieval*, pages 253–260. ACM, 2002.

[19] Benjamin M. Marlin and Richard S. Zemel. Collaborative prediction and ranking with non-random missing data. In *RecSys*, 2009.

[20] Yehuda Koren. Factorization Meets the Neighborhood: a Multifaceted Collaborative Filtering Model. In *KDD*, page 9, 2008.

[21] Maciej Kula. *Spotlight*. GitHub, 2017.

[22] Nicolas Hug. *Surprise, a Python library for recommender systems*. 2017.

[23] T. George and S. Merugu. A Scalable Collaborative Filtering Framework Based on Co-Clustering. In *Fifth IEEE International Conference on Data Mining (ICDM'05)*, pages 625–628, Houston, TX, USA, 2005. IEEE.

[24] Daniel Lemire and Anna Maclachlan. Slope One Predictors for Online Rating-Based Collaborative Filtering. *arXiv:cs/0702144*, February 2007. arXiv: cs/0702144.

[25] Fabian Pedregosa, Gaël Varoquaux, Alexandre Gramfort, Vincent Michel, Bertrand Thirion, Olivier Grisel, Mathieu Blondel, Peter Prettenhofer, Ron Weiss, Vincent Dubourg, and others. Scikit-learn: Machine learning in Python. *Journal of Machine Learning Research*, 12(Oct):2825–2830, 2011.

[26] Leo Breiman. Random forests. *Machine learning*, 45(1):5–32, 2001.
